# Time-of-flight MRA of intracranial vessels at 7 T

**DOI:** 10.1186/s41747-024-00463-z

**Published:** 2024-06-07

**Authors:** Mirco Cosottini, Tommaso Calzoni, Guido Andrea Lazzarotti, Alessandro Grigolini, Paolo Bosco, Paolo Cecchi, Michela Tosetti, Laura Biagi, Graziella Donatelli

**Affiliations:** 1 Department of Translational Research on New Technologies in Medicine and Surgery, University of Pisa, Pisa, Italy; 2 Neuroradiology Unit, Azienda Ospedaliero-Universitaria Pisana, Pisa, Italy; 3 Laboratory of Medical Physics and Magnetic Resonance, IRCCS Stella Maris, Pisa, Italy; 4 Imago7 Research Foundation, Pisa, Italy

**Keywords:** Angiography (digital subtraction), Central nervous system vascular malformations, Magnetic fields, Magnetic resonance angiography, Primary angiitis of the central nervous system

## Abstract

**Background:**

Three-dimensional time-of-flight magnetic resonance angiography (TOF-MRA) is a largely adopted non-invasive technique for assessing cerebrovascular diseases. We aimed to optimize the 7-T TOF-MRA acquisition protocol, confirm that it outperforms conventional 3-T TOF-MRA, and compare 7-T TOF-MRA with digital subtraction angiography (DSA) in patients with different vascular pathologies.

**Methods:**

Seven-tesla TOF-MRA sequences with different spatial resolutions acquired in four healthy subjects were compared with 3-T TOF-MRA for signal-to-noise and contrast-to-noise ratios as well as using a qualitative scale for vessel visibility and the quantitative Canny algorithm. Four patients with cerebrovascular disease (primary arteritis of the central nervous system, saccular aneurism, arteriovenous malformation, and dural arteriovenous fistula) underwent optimized 7-T TOF-MRA and DSA as reference. Images were compared visually and using the complex-wavelet structural similarity index.

**Results:**

Contrast-to-noise ratio was higher at 7 T (4.5 ± 0.8 (mean ± standard deviation)) than at 3 T (2.7 ± 0.9). The mean quality score for all intracranial vessels was higher at 7 T (2.89) than at 3 T (2.28). Angiogram quality demonstrated a better vessel border detection at 7 T than at 3 T (44,166 *versus* 28,720 pixels). Of 32 parameters used for diagnosing cerebrovascular diseases on DSA, 27 (84%) were detected on 7-T TOF-MRA; the similarity index ranged from 0.52 (dural arteriovenous fistula) to 0.90 (saccular aneurysm).

**Conclusions:**

Seven-tesla TOF-MRA outperformed conventional 3-T TOF-MRA in evaluating intracranial vessels and exhibited an excellent image quality when compared to DSA. Seven-tesla TOF-MRA might improve the non-invasive diagnostic approach to several cerebrovascular diseases.

**Relevance statement:**

An optimized TOF-MRA sequence at 7 T outperforms 3-T TOF-MRA, opening perspectives to its clinical use for noninvasive diagnosis of paradigmatic pathologies of intracranial vessels.

**Key points:**

• An optimized 7-T TOF-MRA protocol was selected for comparison with clinical 3-T TOF-MRA for assessing intracranial vessels.

• Seven-tesla TOF-MRA outperformed 3-T TOF-MRA in both quantitative and qualitative evaluation.

• Seven-tesla TOF-MRA is comparable to DSA for the diagnosis and characterization of intracranial vascular pathologies.

**Graphical Abstract:**

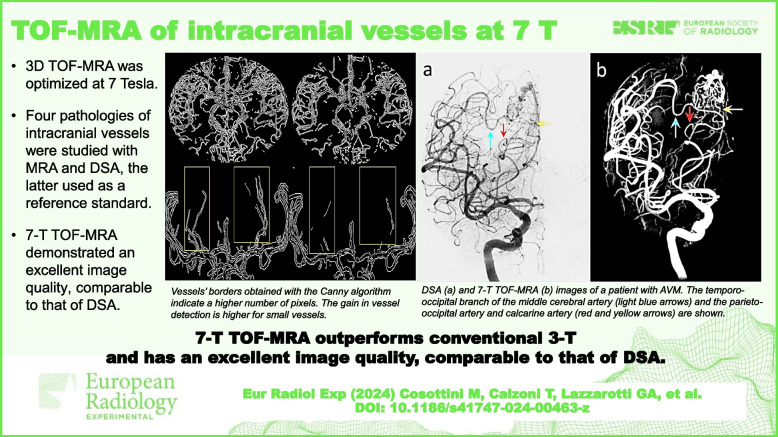

## Background

Cerebrovascular diseases are a main cause of mortality and morbidity, with ischemic and hemorrhagic strokes representing the third cause of death in the world [1]. Imaging has been introduced in guidelines and clinical protocols of both types of strokes as a fundamental diagnostic procedure to identify lesions and cerebrovascular anomalies causing or predisposing to stroke events [2–4].

A large proportion of cerebrovascular diseases affecting intracranial vessels can be studied by imaging the cerebral vasculature. Digital subtraction angiography (DSA) is the reference standard technique for *in vivo* evaluation of cerebral vessels owing to its high spatial and temporal resolution [5]. However, due to the invasive nature of this diagnostic method, non-invasive techniques such as computed tomography angiography and magnetic resonance angiography (MRA) have been affirmed to be appropriate in clinical practice [6]. In studying intracranial vessels, MRA is often adopted because of its non-invasive nature (requiring neither radiation exposure nor intravenous contrast media administration), with time-of-flight (TOF) being the most used technique to depict intracranial arteries. It is a white blood, flow-based MRA technique exploiting the flow-related enhancement: by applying multiple radiofrequency pulses, the intravessels unsaturated spins flowing towards the acquisition plane appear bright with respect to the saturated stationary spins of the surrounding brain parenchyma [7].

Ultra-high field (UHF) scanners, which are approaching use in a clinical environment [8], provide several advantages in imaging the central nervous system [9], including the improvement of TOF-MRA acquisitions [10]. At 7 T, the increased longitudinal relaxation time constant (T1) [11] of the stationary spins promotes their saturation under the radiofrequency pulses of the TOF sequence, increasing the contrast between the flowing spins in the peripheral small vessels and the background. For these reasons, the first attempts to image anatomy and pathology of intracranial arteries have provided promising results [12] in detecting the smallest branches of intracranial vessels [13].

The main constraint of TOF-MRA at UHF is the increase of the specific absorption rate and the potential associated thermal dose that is further worsened by the radiofrequency exposure necessary to cover the entire intracranial arterial tree [14]. However, technical advancements such as high-performant gradients and parallel imaging allow safe acquisition of TOF-MRA covering the entire intracranial vessel anatomy without downgrading sequence parameters [15]. Furthermore, the introduction of compress sensing allows obtaining MRA with a very high spatial resolution in an acceptable acquisition time [16].

In this study, we aimed to explore the performance of MRA obtained with the TOF technique at 7 T. First, we tested our 7-T TOF-MRA sequence on healthy volunteers to optimize the acquisition protocol and confirm that it outperforms the conventional 3-T TOF-MRA. Second, we compared the angiograms obtained with 7-T TOF-MRA in patients with different vascular pathologies with those obtained with DSA as the standard of reference to evaluate their clinical effectiveness.

## Methods

### Seven-tesla TOF-MRA optimization

Four healthy volunteers (two males, two females; aged 33, 40, 39, and 40 years) underwent TOF-MRA using a SIGNA 7-T system (GE Healthcare, Milwaukee, WI, USA) equipped with a 2-channel transmitter/32-channel receiver head coil (Nova Medical, Wilmington, MA, USA).

The sequence had the following parameters: repetition time 12.0 ms; echo time 2.8 ms; flip angle 25°; field of view 200 × 163 mm^2^; 6 slabs with 50% overlap and an overall brain coverage of about 105 mm; and phase acceleration factor (ARC) = 2 compress sensing factor (Hypersense) 1.2. The voxel size was progressively reduced until the signal-to-noise ratio (SNR) reached that obtained with the 3-T TOF-MRA used for clinical acquisitions. Seven-tesla TOF-MRA sequences were acquired at three different spatial resolutions:

i) Matrix size 400 × 400, slice thickness 1 mm, and images reconstructed with a voxel size of 0.2 × 0.2 × 0.5 mm^3^ (7-T acq1);
ii) Matrix size 500 × 500, slice thickness 0.8 mm, and images reconstructed with a voxel size of 0.2 × 0.2 × 0.4 mm^3^ (7-T acq2);
iii) Matrix size of 660 × 660, slice thickness 0.6 mm, and images reconstructed with a voxel size of 0.2 × 0.2 × 0.3 mm^3^ (7-T acq3).

Each sequence at a specific spatial resolution was acquired twice, without any prescan and shimming optimization in between, in order to calculate the SNR by the difference method [17].

To compare the SNR of 7-T TOF-MRA with that of the clinical 3-T TOF-MRA, a TOF-MRA sequence was acquired in each of the four healthy volunteers using a 3-T system (MR750 scanner, GE Healthcare, Milwaukee, WI, USA) equipped with an 8-channel receiver head coil. The sequence had the following parameters: repetition time 19.0 ms; echo time 2.9 ms; flip angle 20°; field of view 200 × 162 mm^2^; 7 slabs with 50% overlap and an overall brain coverage of 112 mm; phase acceleration factor (ASSET) of 2; matrix size of 416 × 320; slice thickness 1 mm; and images reconstructed with a voxel size of 0.4 × 0.4 × 0.5 mm^3^. As it was for 7 T, also this sequence was acquired twice, without any prescanning and shimming optimization in between, to allow SNR calculation by the difference method.

### Study of intracranial vascular pathologies

**Fig. 1:**
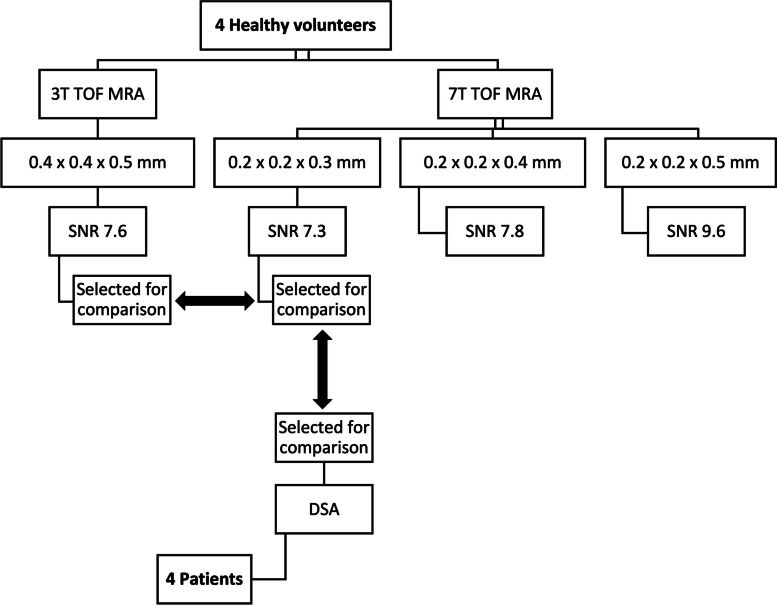
Flowchart of the study. *SNR* Signal-to-noise ratio, *TOF-MRA* Time-of-flight magnetic resonance angiography

We enrolled four patients affected by different types of intracranial vascular pathologies, namely intracranial stenosis in primary arteritis of the central nervous system (PACNS), saccular aneurysm, arteriovenous malformation (AVM), and dural arteriovenous fistula (DAVF). All patients underwent both the optimized 7-T TOF-MRA protocol and the diagnostic DSA (Fig. 1).

The DSA was performed with a GE Healthcare Innova biplane angiograph with local anesthesia and a 6F retrograde femoral access allowing selective catheterization of both internal and external carotid arteries and at least one vertebral artery for studying carotid and vertebrobasilar circulation. In the patient with the intracranial aneurysm, a three-dimensional rotational acquisition was also performed during the selective injection of the affected vessel.

### Image analysis for protocol optimization

For each couple (A, B) of repeated MRI acquisitions (${{\text{TOF}}}_{i}^{A}$, ${{\text{TOF}}}_{i}^{B}$, with $i=1,\dots ,4$; where $i=1,\dots , 3$ indicates the three 7-T acquisitions at different spatial resolution and $i=4$ refers to the acquisition at 3 T), ${{\text{TOF}}}_{i}^{B}$ was registered to ${{\text{TOF}}}_{i}^{A}$, through the Advanced Normalization Tools−ANTs [18]. The registration scheme consisted in a multiscale 6 degrees-of-freedom rigid transform optimizing mutual information similarity metric and applying a nearest neighbor criterion to prevent from interpolation smoothing.

The ${{\text{TOF}}}_{i}^{A}$ datasets (for *i* = 1, 2, and 4), were then registered to the 7-T dataset with the highest spatial resolution (${{\text{TOF}}}_{3}^{A}$) by following the very same registration scheme. To avoid multiple interpolations, the two consecutive transforms needed to reach the highest resolution space were calculated separately, combined, and then applied just once to reach the native space. From this registration phase, the inverse transforms were also calculated.

On the highest resolution 7-T acquisition (${{\text{TOF}}}_{3}^{A}$), 17 ROIs were manually segmented by a radiologist and the ROIs were reported on the lower resolutions native space by applying the inverse transforms. ROIs were placed within intracranial vessels of large (carotid siphon, basilar artery), medium (sphenoidal segment of the middle cerebral artery—M1, pre-communicating segment of the anterior cerebral artery—A1, pre-communicating segment of the posterior cerebral artery—P1), and small (insular segment of the middle cerebral artery—M2, post-communicating segment of the anterior cerebral artery—A2, post-communicating segment of the posterior cerebral artery—P2) diameter.

For the measurement of CNR, for each ROI, a parenchymal ${{\text{ROI}}}^{\mathrm{^{\prime}}}$ of the same size was placed in the parenchymal tissue adjacent to the vessel of interest, in order to estimate the difference in signal intensity between the vessel and the closest parenchyma.

For each ${{\text{ROI}}}_{j}$ $(j$= 1, …, 17) and each acquisition scheme $i$ ($i$= 1, …, 4), the SNR was estimated by using the following formula:

$${{\text{SNR}}}_{i, {{\text{ROI}}}_{j}}=\frac{1}{\sqrt{2}} \frac{{{\text{mean}}}_{{{\text{ROI}}}_{j}}({{\text{TOF}} }_{i}^{A} , { {\text{TOF}} }_{i}^{B})}{{\sigma }_{{{\text{ROI}}}_{j}}({{\text{TOF}} }_{i}^{A}- {{\text{TOF}} }_{i}^{B})}$$

where the numerator corresponds to the mean of images A and B, calculated on the *j*th ROI, and the denominator is the standard deviation, calculated on the *j*th ROI, of the difference image of A and B.

Analogously, for each ${{\text{ROI}}}_{j}$ $(j$= 1, …, 17) and each acquisition scheme $i$ ($i$= 1, …, 4), the CNR was estimated according to the formula:

$${{\text{CNR}}}_{i, {{\text{ROI}}}_{j}}=\frac{{{\text{TOF}} }_{i, {{\text{ROI}}}_{j} }^{A}- {{\text{TOF}} }_{i, {{\text{ROI}}}_{j}{\prime} }^{A} }{\sqrt{{\sigma ({{\text{TOF}} }_{i}^{A})}_{{{\text{ROI}}}_{j}}^{2}+ {\sigma ({{\text{TOF}} }_{i}^{A})}_{{{\text{ROI}}}_{j}{\prime}}^{2}}}$$

where the numerator is the signal difference between the *j*th ${ROI}_{j}$ and the corresponding *j*th parenchymal ROI (${{\text{ROI}}}_{j}{\prime}$), whereas the denominator is the root sum squared of their standard deviations.

The optimized 7-T TOF-MRA protocol at the highest resolution was compared with the conventional 3-T TOF-MRA using a qualitative and a quantitative method.

The qualitative evaluation was conducted by a neuroradiologist with 23 years of experience who graded the vessel visibility using a 5-point scale ranging from 0 to 4 (0, vessel not visible; 1, poor vessel visibility; 2 satisfactory visibility; 3, good visibility; 4, excellent visibility). The neuroradiologist was invited to inspect and grade the following intracranial vessels: carotid siphon, ophthalmic artery, lenticulostriate arteries, anterior choroidal artery, thalamic perforating arteries, pontine perforating arteries, anterior cerebral arteries, middle cerebral arteries, posterior cerebral arteries.

The quantitative evaluation of the angiogram’s quality was performed with the automatic detection of vessels’ borders, based on the Canny algorithm [19], and implemented in the software ImageJ (National Institute of Health, Bethesda, USA). This evaluation was conducted on axial maximum intensity projection (MIP) images (containing vessels of all sizes) and on isolated lenticulostriate arteries images (representing small vessels).

### Image analysis for studying intracranial vascular pathologies

The evaluation of the intracranial vascular pathologies was performed by a neuroradiologist with 18 years of experience on the angiograms obtained with both MRA and DSA and included the measurement of radiological parameters adopted for diagnosis and therapeutical planning. All TOF-MRA data sets were transferred to a dedicated workstation (Advantage Workstation Volume Share 2, GE Healthcare, Milwaukee, WI, USA) and reconstructed with multiplanar volume reconstruction and volume rendering algorithms. Conventional two-dimensional and three-dimensional antero-posterior, latero-lateral, right-left oblique projections were obtained and saved for qualitative comparison with the homologous angiograms obtained with DSA. In the case of PACNS, the number of stenosis was calculated. In the case of aneurysm, the diameters of the aneurysmal dome, neck, and parent vessel were measured with an electronic caliper. An automatic stent deployment simulation was performed using the software Ankyras (Galgo Medical, Barcelona, Spain) and the stent parameters were compared. In the case of AVM, the diameter of the nidus, the number of feeders, and their origins were recorded. In the case of DAVF, the number of feeders and their origins were recorded.

The quantitative evaluation of the image similarity between 7-T TOF-MRA and DSA was performed by calculating the complex-wavelet structural similarity index (CW-SSI) [20] between two corresponding images. For MRA, a MIP image was used. For DSA, a standard two-dimensional image was used and substituted by a MIP image when a three-dimensional acquisition was available. Both the images undergoing comparison were first segmented using the Otsu method [21]. Given two images $x$ and $y$, the CW-SSI was computed as follows:

$$\text{CW-SSI}\left({c}_{x},{c}_{y}\right)=\frac{2\left|{\sum }_{1=1}^{N}{c}_{x,i}{c}_{y,i}^{*}\right|+K}{{\sum }_{i=1}^{N}{\left|{c}_{x,i}\right|}^{2}+{\sum }_{i=1}^{N}{\left|{c}_{y,i}\right|}^{2}+K}$$

where ${c}_{x,i}$ and ${c}_{y,i}$ are two complex wavelet transform coefficients extracted from the same sub-band at the same location. $N$ represents the total number of coefficients in a specific sub-band. The parameter $K$ is a constant, which improves the evaluation of the CW-SSI in case of low SNR. This similarity index is insensitive to small relative rotations and translations of the images, this is evident when expressed as:

$$\text{CW-SSI}\left({c}_{x},{c}_{y}\right)=\frac{2{\sum }_{i=1}^{N}\left|{c}_{x,i}\right|\left|{c}_{y,i}\right|+K}{{\sum }_{i=1}^{N}{\left|{c}_{x,i}\right|}^{2}+{\sum }_{i=1}^{N}{\left|{c}_{y,i}\right|}^{2}+K}\cdot \frac{2\left|{\sum }_{i=1}^{N}{c}_{x,i}{c}_{y,i}^{*}\right|+K}{2{\sum }_{i=1}^{N}\left|{c}_{x,i}{c}_{y,i}^{*}\right|+K}$$

Considering two identical images *x* and *y* with a relative spatial translation between them, the coefficient ${c}_{y,i}$ will be well approximated by a phase-shifted version of the corresponding coefficient ${c}_{x,i}$. It follows that the first term of the product, which is dependent on the magnitude of the coefficients, will be 1. The second term of the product is dependent on the consistency of the phase shifts between two corresponding wavelet transform coefficients for all $i$. A small translation will cause a constant phase shift of ${c}_{y,i}$ with respect to the corresponding coefficient ${c}_{x,i}$ for all $i$, causing the second term of the product to be 1. Small rotations can be considered as small translations so that what is expressed above remains valid. A translation is considered small with respect to the wavelet filter size and its envelope decay.

## Results

### Acquisition protocol optimization

The mean specific absorption rate value averaged over 6 min of scan was less than 3.2 W/kg for all the 7-T MRA acquisitions.

**Fig. 2:**
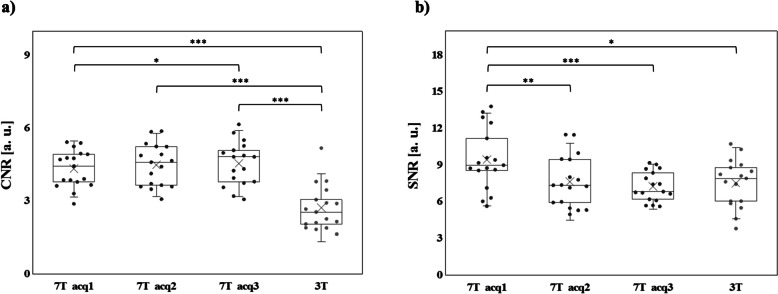
CNR (**a**) and SNR (**b**) calculated for the three 7-T TOF-MRA sequences at different spatial resolutions (black color) and for the clinical 3-T TOF-MRA images (dark gray color). Each dot represents the mean among subjects of CNR (**a**) or SNR (**b**) for a specific ROI. Data distribution is shown through box plots, where the box is determined by the 25^th^ and 75^th^ percentiles and the median and the mean are represented by the horizontal and the cross line inside the box, respectively. The whiskers represent the 5^th^ and 95^th^ percentiles. To compare results for CNR (**a**) or SNR (**b**) obtained by different acquisitions, nonparametric paired tests (Wilcoxon signed-rank test) were performed between each couple of datasets. Statistically significant differences of CNR and SNR between different acquisition schemes are reported in the graph according to the following legend: one asterisk (*) indicates *p* < 0.050, two asterisks (**) *p* < 0.010, and three asterisks (***) *p* < 0.001. *CNR* Contrast-to-noise ratio, *SNR* Signal-to-noise ratio, *TOF-MRA* Time-of-flight magnetic resonance angiography

The mean CNR, averaged among ROIs and subjects, was significantly higher at 7 T than at 3 T (CNR_7T = 4.5 ± 0.8 *versus* CNR_3T = 2.7 ± 0.9, *p* < 0.001) irrespective of the spatial resolution of 7-T TOF-MRA (Fig. 2a). The SNR of 7-T MRA decreased with reducing voxel size (Fig. 2b). The mean SNR of all intracranial vessels reached the value of 7.3 when measured on MRA with a reconstructed voxel size of 0.2 × 0.2 × 0.3 mm, which was immediately lower than that (7.5) obtained at 3 T. This optimized 7-T TOF-MRA protocol (7-T acq3; scan time 10:43 min/s) was used for all the applications and comparisons in the present study.

The image quality score in the four volunteers is reported in Table 1. The mean quality score for all vessels was higher at 7 T (2.89) than at 3 T (2.28). While the largest intracranial vessels have a comparable image quality (excellent at both 3 and 7 T), the smallest intracranial vessels (lenticulostriate arteries and thalamic perforating arteries) have a higher mean score at 7 than at 3 T (3.50 *versus* 1.63, respectively). A poor-quality score was reached in evaluating the pontine perforating arteries (1 at 7 T and 0 at 3 T) and the ophthalmic arteries (2.63 at 7 T and 3.00 at 3 T).

**Table 1:** Image quality scores of 7-T and 3-T time-of-flight magnetic resonance angiography in four healthy volunteers

Vessels	Volunteer 1		Volunteer 2		Volunteer 3		Volunteer 4	
	7 T	3 T	7 T	3 T	7 T	3 T	7 T	3 T
Carotid siphons	3	4	3	3	3	3	3	3
Ophthalmic arteries	3	4	4	3	1	1	1	3
Lenticulostriate arteries	4	3	4	2	4	3	4	3
Anterior choroidal arteries	3	3	3	3	3	3	3	2
Thalamic perforating arteries	4	2	3	0	2	0	3	0
Pontine perforating arteries	0	0	2	0	1	0	1	0
Anterior, middle, and posterior cerebral arteries	4	4	4	4	4	4	4	4
Total score	21	20	23	15	18	14	19	15

**Fig. 3:**
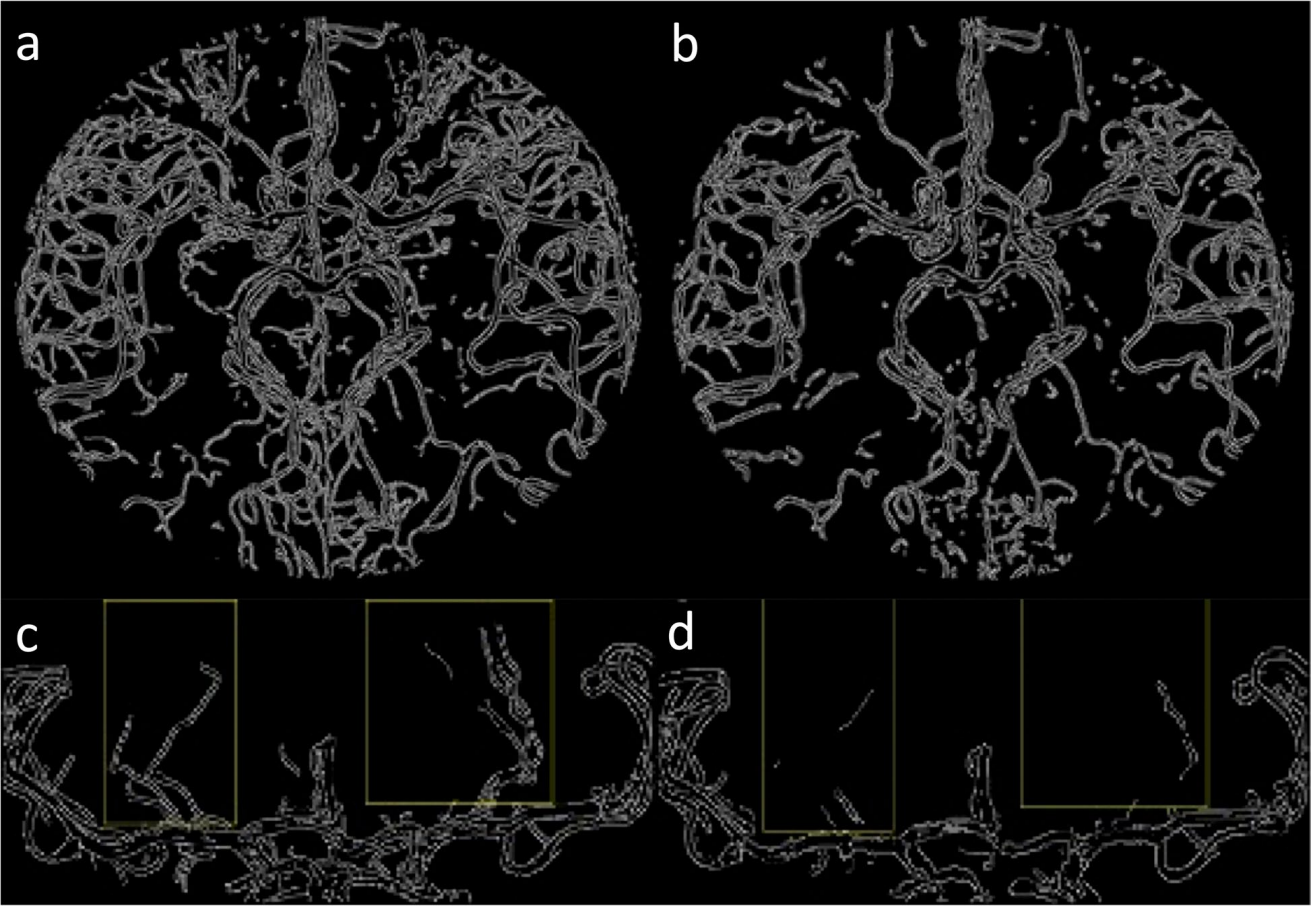
Graphical representation of the pixels pertaining to the vessels’ borders obtained with the Canny algorithm on the axial maximum intensity projection images at 7 T (**a**) and 3 T (**b**), and on the lenticulostriate arteries (within the boxes) imaged at 7 T (**c**) and 3 T (**d**)

The quantitative evaluation of the angiogram’s quality using the Canny algorithm revealed a higher vessel border detection at 7 T than at 3 T (44,166 *versus* 28,720 pixels for the axial MIP images, and 2,766 *versus* 480 pixels for the isolated lenticulostriate arteries) (Fig. 3).

### Evaluation of intracranial vascular pathologies

The comparative radiological parameters used for the diagnosis and characterization of intracranial vascular pathologies with 7-T TOF-MRA and DSA are reported in Table 2.

**Table 2:** Radiological diagnostic parameters used for the comparative evaluation of the intracranial pathologies

**Pathology**	**Radiological parameters**
Primary arteritis of the central nervous system	Number of stenosis
Aneurysm	Measurements of dome, neck, and parent vessel diameter
	Automatic stent placement simulation
Arteriovenous malformation	Nidus dimensions
	Number of identified feeders
	Origins of the feeders
Dural arteriovenous fistula	Number of identified feeders
	Origins of the feeders

#### PACNS

**Fig. 4:**
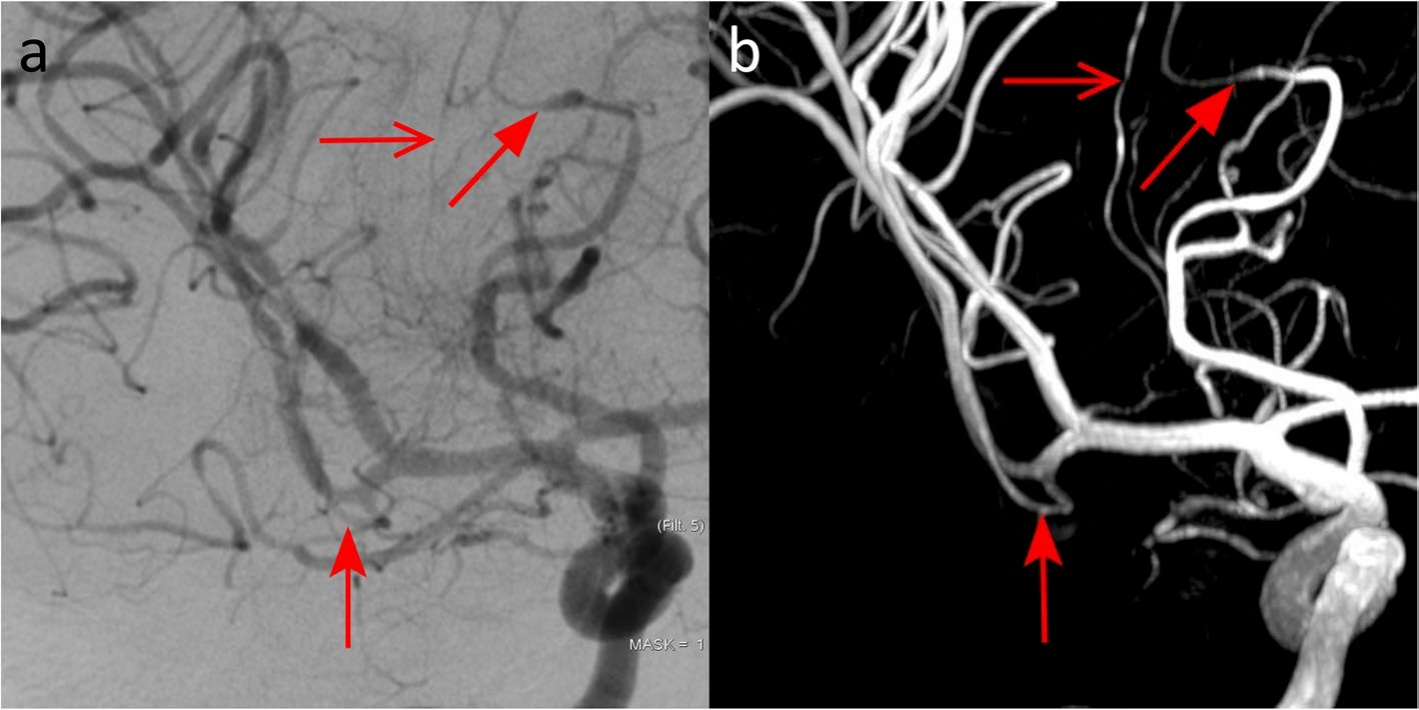
Digital subtraction angiography (**a**) and 7-T TOF-MRA (**b**) images in the patient with primary arteritis of the central nervous system. The selective contrast injection of the right internal carotid artery revealed multiple segments of vessels narrowing on both anterior and middle cerebral arteries: most of these stenoses (arrows) were confirmed in the multiplanar volume reconstruction of 7-T TOF-MRA (**b**). *TOF-MRA* Time-of-flight magnetic resonance angiography

In the patient with intracranial stenosis due to PACNS (Fig. 4), the 7-T TOF-MRA revealed 16 intracranial stenosis out of 19 detected with DSA (84.2%). The quantitative comparison of 7-T TOF-MRA and DSA angiograms revealed a CW-SSI of 0.74.

#### Aneurysm

**Fig. 5:**
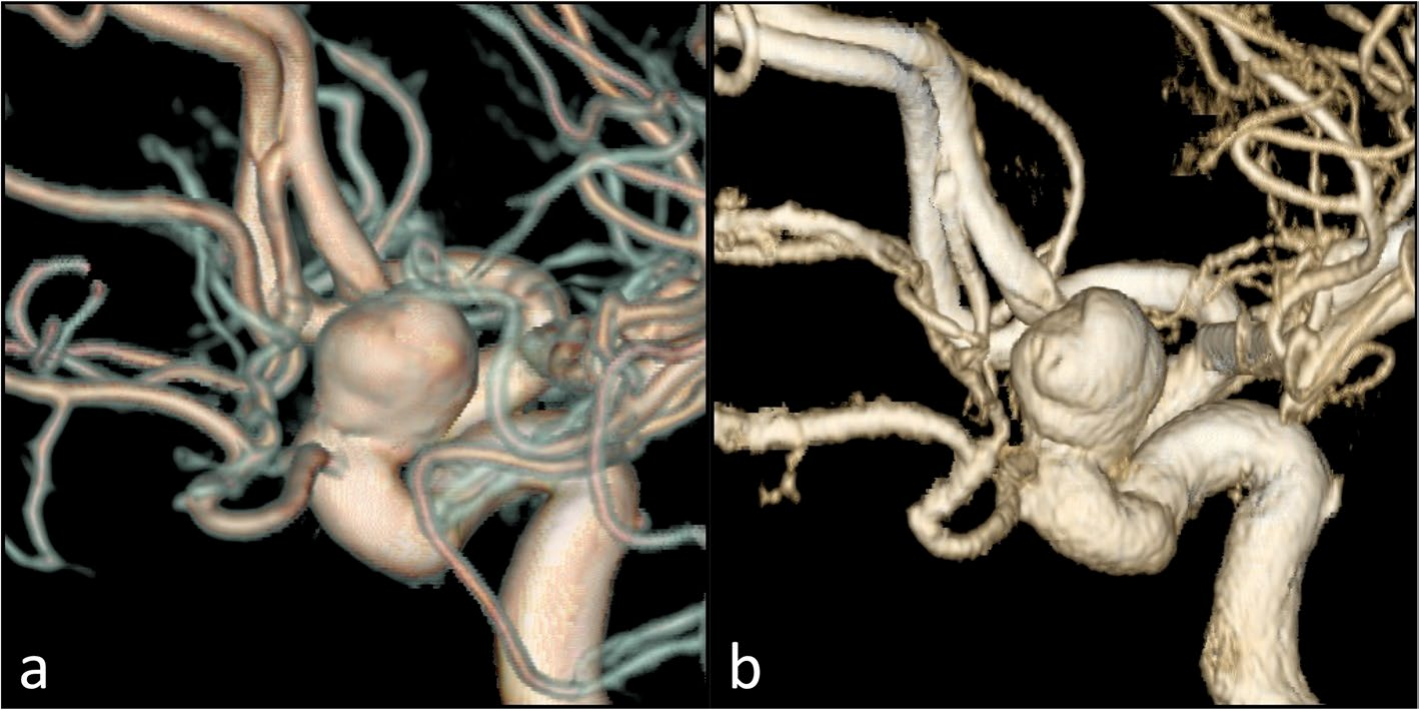
DSA (**a**) and 7-T TOF-MRA (**b**) in the patient with carotid artery aneurysm. Three-dimensional reconstruction of a rotational DSA with images acquired during the selective injection of the left internal carotid artery (**a**) showed the presence of a saccular aneurysm of the ophthalmic segment of the internal carotid artery. The volume rendering of the 7-T TOF-MRA allowed an optimal depiction of the aneurysmal geometric characteristics (**b**). *DSA* Digital subtraction angiography, *TOF-MRA* Time-of-flight magnetic resonance angiography

**Fig. 6:**
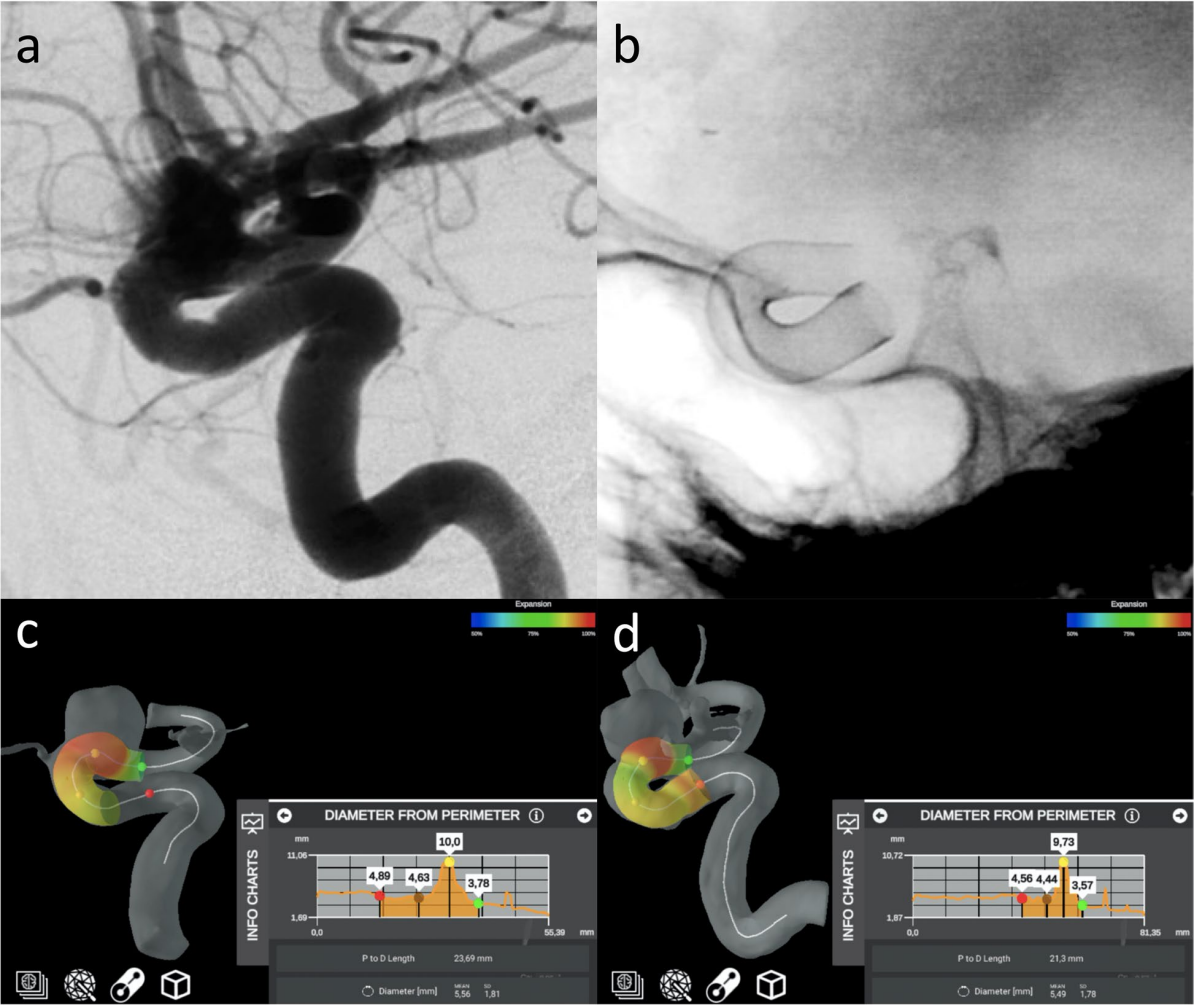
Intraprocedural DSA angiograms of the stent placement (**a**, **b**) and the stent placement simulations obtained with an automatic software (Ankyras, Galgo Medical, Barcelona, Spain) from the DSA (**c**) and 7-T TOF-MRA dataset (**d**). The red, brown, yellow, and green spherical markers indicate the spatial location of the parameter value shown in the side graph. *DSA* Digital subtraction angiography, *TOF-MRA* Time-of-flight magnetic resonance angiography

In the patient with paraophthalmic aneurysm (Fig. 5), the diameters of aneurismal neck and dome measured on 7-T TOF-MRA and DSA images were comparable. The dome measured 8.5 × 9.3 × 7.6 mm on the 7-T MRA and 8.4 × 9.3 × 7.5 mm on DSA (craniocaudal × anteroposterior × latero-lateral). The aneurysmal neck measured 6.7 × 7.9 mm on 7-T TOF-MRA and 6.9 × 7.8 mm on DSA (antero-posterior × latero-lateral). The average difference between the two corresponding measures is 0.07 ± 0.06 mm (mean ± standard deviation). The measurements in the proximal and distal segment of the parent vessel with an electronic caliper were respectively 4.2 and 3.4 mm on 7-T TOF-MRA and 4.0 and 3.6 mm on DSA. Moreover, the automatic stent placement simulation gave a correct prediction of the actual stent expansion and placement, yielding comparable results between angiographic methods (Fig. 6). From the simulation, the parent vessel proximal and distal diameters were respectively 4.6 mm and 3.6 mm for the 7-T TOF-MRA dataset and 4.9 mm and 3.8 mm for the DSA dataset; the vessel diameter at the aneurysm was 9.7 mm for the 7-T TOF-MRA data set and 10.0 mm for the DSA data set and the difference between two corresponding measures from the two data sets was 0.26 ± 0.11 mm. The quantitative comparison of 7-T TOF-MRA and DSA in evaluating the aneurismatic vessel revealed a CW-SSI of 0.90.

#### AVM

**Fig. 7:**
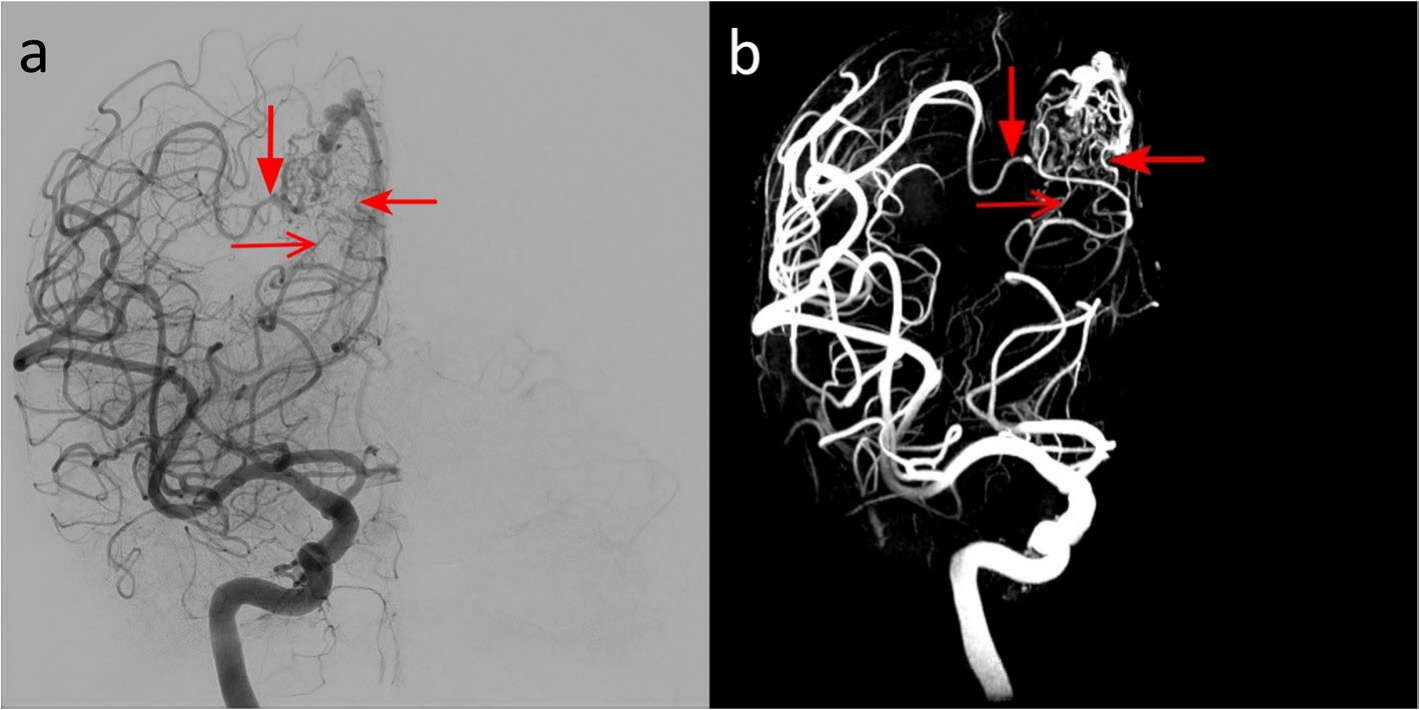
DSA (**a**) and 7-T TOF-MRA (**b**) images of the patient with plessiform AVM of the occipital lobe. The AVM had a superficial nidus fed by arterial afferents arising from carotid and vertebral circulations. The DSA angiogram obtained by the selective injection of the right internal carotid artery (**a**) showed multiple feeders from the temporo-occipital artery arising from the middle cerebral arteries (filled arrow). In this patient, being the posterior cerebral artery supplied by the carotid circulation via the posterior communicating artery, some afferents from the parieto-occipital artery and calcarine artery are also opacified (open arrow and dented arrows)

In the patient with AVM in the occipital lobe, the nidus dimension was found to be 20 mm with both the 7-T TOF-MRA and the DSA. The number of identified feeders was 4 for both the modalities (Fig. 7). The feeders were found to originate from the right angular artery, the right temporo-occipital artery, the right parieto-occipital artery, and the right calcarine artery. The quantitative comparison of 7-T TOF-MRA and DSA revealed a CW-SSI of 0.76.

#### DAVF

**Fig. 8:**
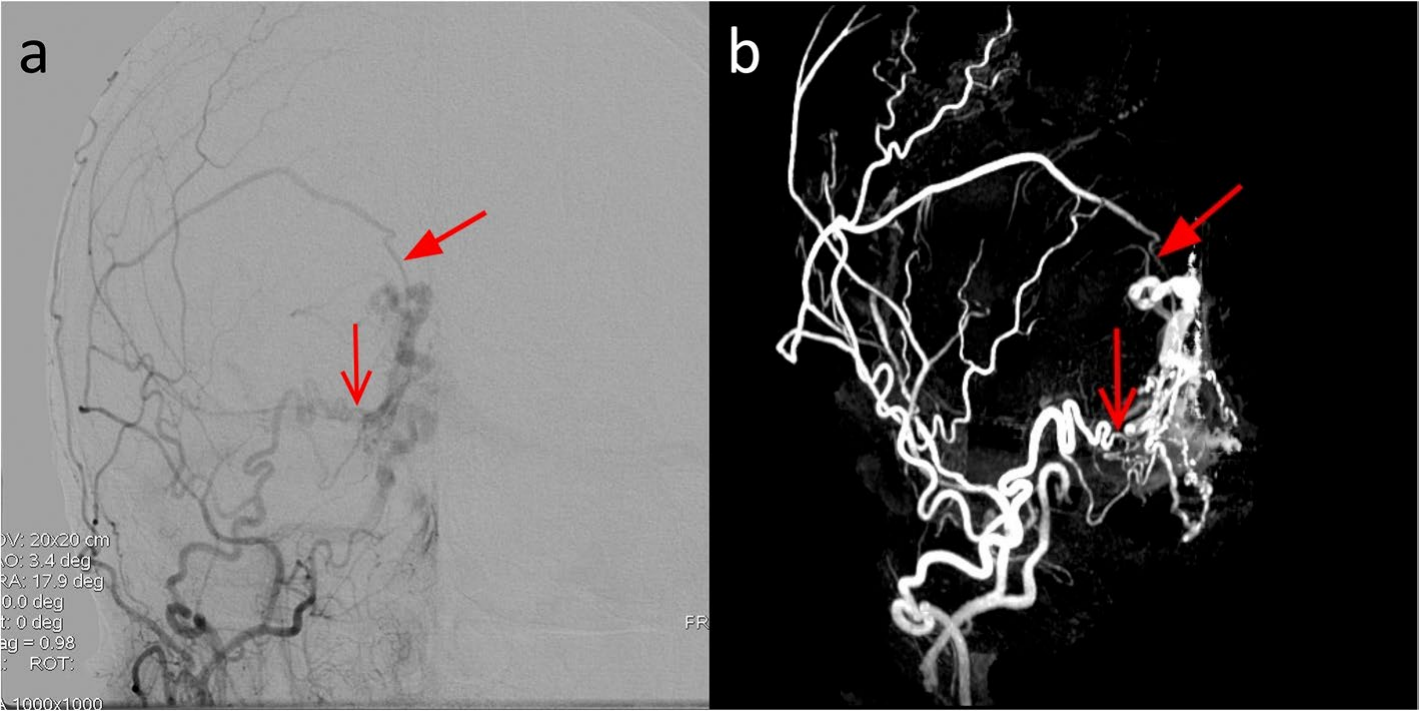
DSA (**a**) and 7-T TOF-MRA (**b**) in the patient with DAVF type III of Cognard. The selective injection of the right external carotid arteries (**a**) depicted meningeal feeders arising from the right middle meningeal artery (light blue arrows) and from the right occipital artery (red arrow)

In the patients with DAVF type III of Cognard, five feeders were identified on both modalities (Fig. 8). The feeders were found to originate from the left posterior meningeal artery, the left middle meningeal artery, the left occipital artery, the right middle meningeal artery, the right occipital artery, and the right ascending pharyngeal artery. The quantitative comparison of 7-T TOF-MRA and DSA angiograms revealed a CW-SSI of 0.52.

## Discussion

In our study, the higher SNR at 7 T allows to optimize the 7-T TOF-MRA by increasing the spatial resolution (up to 0.2 × 0.2 × 0.3 mm^3^) until the SNR reached that of a clinical 3-T TOF-MRA in a tolerable and clinically feasible acquisition time (about 10 min) [22]. The improved spatial resolution of the 7-T TOF-MRA allowed a high-quality depiction of the intracranial vessels’ normal anatomy. Both qualitative and quantitative evaluation of 7-T angiograms demonstrated an improvement of vessel depiction with respect to conventional clinical 3-T acquisitions. Specifically, the highest diagnostic gain was obtained in depicting the distal branches of dichotomy of intracranial vessels and small vessels such as lenticulostriate arteries and thalamic perforating arteries. The improvement of the 7-T TOF-MRA in revealing the thin peripheral vessels is related to the more efficient background suppression typical of the UHF scanners [23] and to the use of smaller voxels which reduces the partial volume effects occurring when the vessel is off-centered in the voxel. Moreover, the availability of all the classical methods for increasing the flow sensitivity of TOF-MRA, such as ramped radiofrequency pulses with flip angles varying as a function of position [24], fat suppression pulses, and the Multiple Overlapping Thin Slab Acquisition−MOTSA technique [25], improves the quality of the vessel imaging at UHF. Notwithstanding 7-T TOF-MRA might reach an unprecedented spatial resolution allowing the *in vivo* visualization of pial arteries [26], in our study the visualization of pontine perforating arteries remains poor because of their very small diameter (less than 0.3 mm) [10]. An improvement in depicting these perforating arteries has been demonstrated by using the TOF sequence after contrast media administration [13]. Similarly, the visualization of the origin of the ophthalmic artery is suboptimal presumably due to the high sensitivity to magnetic susceptibility phenomena and the vicinity with the paranasal sinuses. The substantial qualitative similarity between 7 T and 3 T in imaging most proximal vessels (carotid siphons and basilar artery) can be due to the higher dielectric effect at UHF. Although the use of dielectric pads [27] and B_1_ shimming [28] are suggested to limit this physical effect, the less effectiveness of the transmit coil used at 7 T with respect to the body coil used at 3 T might prevent the full exploiting of UHF in this vascular district.

The long acquisition time of TOF-MRA is reduced in our protocol by using parallel imaging and optimized under-sampling parameters, allowing the acquisition of the entire vessels’ anatomy in 10:43 min:s and the mitigation of the specific absorption rate level which is maintained within the prescribed safety limits [29].

The extraordinary level of anatomical detail of 7-T TOF-MRA is comparable to that of the DSA and confirms previous experiences in the detection of small arteries such as the lenticulostriate arteries [30], the perforating arteries originating from the posterior communicating artery, or the microanatomy and course of the subcallosal artery [31].

Concerning the different vascular pathologies, the comparison with DSA indicates that 7-T TOF-MRA has a high accuracy for diagnosis and provides a remarkable global evaluation of the pathological details. Most of the radiological parameters used for diagnosing cerebrovascular diseases on DSA were detected also with 7-T TOF-MRA (27 out of 32, 84%). In particular, in the evaluation of intracranial stenosis, our results were comparable to those obtained with DSA. The assessment of intracranial atherosclerosis has been reported to improve at 7 T with respect to 3 T for both vessel visibility and number of detectable lesions [32]; in the case of moya-moya disease, 7-T TOF-MRA is comparable to DSA even in depicting collateral network pathways [33]. PACNS are characterized by concentric vessel wall thickening of short arterial segments, inducing stenosis alternating with vessel enlargement (beading) [34]. MRA is the method of choice for the non-invasive diagnostic work-up, and 3-T MRA demonstrated a higher diagnostic accuracy with respect to 1.5 MRA [35]. Our 7-T results seem to improve the performance of MRA revealing 84% of the distal stenosis detected with DSA. The implementation of the intracranial vessel wall imaging with 7-T MRA in PACNS is still unexplored [36], but probably it will further increase the positive predictive value of MRA in the non-invasive diagnosis of this disease.

In the patient with intracranial aneurysm, the dimension of the dome and the dimension and position of the aneurysmal neck from the 7-T TOF-MRA corresponded perfectly to those derived from DSA. This justifies the high CW-SSI and it is in agreement with the largest study on aneurysms at 7 T, in which MRA and DSA were qualitatively compared [37]. The stent placement simulation based on the automatic measurements of the aneurysmal and vessel geometry provided similar results between the 7-T MRA and DSA datasets. This could imply that the embolization with stents or spirals could be planned directly on MRA images without requiring a preprocedural DSA acquisition. In this field of application, MRA combined with the high-resolution vessel’s wall imaging at 7 T, could open unexplored scenarios in studying aneurysmal formation [38] or instability, identifying microstructural changes of the aneurysmal wall that are not visible at lower spatial resolutions [39].

In AVM, 7-T TOF-MRA and DSA provided similar information on a number of feeders and their origin from intracranial vessels. In addition, the smallest feeders arising from middle and posterior cerebral arteries were identified as suppliers of the nidus, confirming the excellent image quality and the potential clinical application of ultra-high-field MRA [40]. Similarly, the 7-T angiograms related to the DAVF revealed fine details and allowed identifying of most meningeal arterial feeders. However, TOF-MRA is a static technique, therefore it does not contain information about the blood flow dynamic that is essential for studying and classifying these diseases. The poor visualization of venous drainage dynamic in MRA and the decreased CW-SSI computed for the image pairs representing pathology with strong time dependence (such as DAVF and AVM) confirm this limitation and the need of time-resolved techniques for a more detailed evaluation of this pathology.

Recently, the Society of Magnetic Resonance Angiography [41] summarized pros and cons of 7-T MRA and tried to identify the vascular pathologies for which UHF could have an added clinical value. We optimized the 7-T MRA sequence to obtain a very high spatial resolution in a reasonable acquisition time. However, the 7-T MRA protocol could be adjusted on the basis of specific clinical questions. Indeed, differently from small vessel diseases or vascular malformations, we hypothesize that the evaluation of large aneurysms of the Willis’ circle does not need an extreme spatial resolution, and the 7-T MRA protocol that we proposed can be intended as a compromise to explore most intracranial vascular pathologies in a coming clinical setting.

The main limitation of this study is that the visual and quantitative analysis of the image quality of 7-T TOF-MRA was limited to a case series and our results need to be confirmed in large cohorts of patients for each pathology. Nevertheless, the quantification of the similarity of optimized MRA images with the reference standard DSA indicated a promising role of 7-T TOF-MRA as a technique to investigate intracranial vascular pathology.

Our study focused on TOF-MRA but the possibility to explore intracranial vessels at UHF combining other techniques such as the high-resolution arterial vessel wall imaging with black blood techniques, susceptibility-weighted venography, time-resolved contrast-enhanced MRA, and arterial spin labeling perfusion, which are under investigation at 7 T [42], might improve the non-invasive diagnostic approach to several cerebrovascular diseases.

## Data Availability

Data may be provided to interested researchers upon request to the corresponding author, after clearance from the Ethics Committee.

## Abbreviations

AVM: Arteriovenous malformation
CNR: Contrast-to-noise ratio
CW-SSI: Complex wavelets structural similarity index
DAVF: Dural arteriovenous fistula
DSA: Digital subtraction angiography
MIP: Maximum intensity projection
MRA: Magnetic resonance angiography
PACNS: Primary arteritis of the central nervous system
SNR: Signal-to-noise ratio
TOF: Time-of-flight
UHF: Ultra-high field
